# Comparison of maternal and neonatal survival exposed to humidifier disinfectants during perinatal periods: a case-series study

**DOI:** 10.1038/s41598-023-47438-5

**Published:** 2023-11-16

**Authors:** Jang Hoon Lee, Joon Sung Joh, Seoheui Choi

**Affiliations:** 1 Department of Paediatrics, Ajou University School of Medicine, 164 World Cup-ro, Yeongtong-gu, Suwon, 16499 Republic of Korea; 2 Department of Pulmonology, National Medical Center, Seoul, Republic of Korea

**Keywords:** Environmental sciences, Medical research

## Abstract

A humidifier disinfectant (HD) has been prohibited by the government due to its serious effects on the human body. Several studies on the relationship between HD and lung diseases have been performed independently on children and adults. However, there have been no reports on the effects of HD exposure on pregnant women and their foetuses. Therefore, the present study was conducted to investigate the effects of HD exposure on the foetuses of women who encountered HD during pregnancy. A total of 56 cases were recruited from 2017 to 2019 through the Korea Environmental Industry & Technology Institute, and data obtained from the medical records included maternal date of birth, maternal date of death, maternal start and end date of HD exposure, maternal date of symptom onset, neonatal birthday, neonatal birthweight, gestational age, and neonatal survival status within 28 days. All data were retrospectively investigated through medical records. Of the 47 mothers, 20 (42.6%) mothers survived, and 27 (57.4%) mothers died. In the group of survivors, there was a shorter period of total HD use, period of HD use before pregnancy and period of HD use to onset of symptoms. Shorter durations of HD use resulted in higher survival rate of mothers. HD use caused an increase in gestational age surviving foetuses, and foetal mortality increased when clinical symptoms developed before birth.

## Introduction

In the Republic of Korea, a humidifier disinfectant (HD), which was developed to replace the cleaning of humidifiers mainly used in dry winters, had been on the market since 1994. HD, which contained polyhexamethylene guanidine phosphate (PHMG), oligo (2-(2-ethoxy) ethoxyethyl guanidinium (PGH), or a mixture of chloromethylisothiazolinone (CMIT) and methylisothiazolinone (MIT), was used by individuals who wanted to keep the inside of their humidifier constantly clean^1^.

It has been reported that the cause of interstitial lung disease (ILD) of unknown cause, which has gradually increased since 2006, is HD^2,3^. Due to this disease, named HD lung injury (HDLI), the use of HDs was banned by the government of the Republic of Korea in 2011. In addition, the government has established an HDLI Investigation and Decision Committee to clinically evaluate registered patients who were presumed to have lung injuries due to the use of HDs and to determine whether the damage is clinically related to the use of HDs. Additionally, the HD Extrapulmonary Injury Review Committee was formed in 2017 to determine the effect of HDs on pregnant women. To date, the Korea Centers for Disease Control and Prevention continues retrospective epidemiologic investigations^4,5^.

In a survey conducted on randomly selected 1577 children born between April and July 2008 and turning 7 years old in 2015, 75.6% of children responded that they had used a humidifier. Additionally, 31.1% of these children reported using HD^6^. A total of 63.5% of HDLI cases were found among preschool children under 6 years of age who participated in the HD damage assessment, and 77.1% of HDLI cases were infants under 3 years of age^7^. As such, several studies on the relationship between HD use and lung diseases have been performed by separating children from adults, but there have been no reports on the effects of HD use on pregnant women and their foetuses. Therefore, the present study was conducted to investigate the effects of HDs on the foetuses of women who used HDs during pregnancy.

## Materials and methods

### Study participants

The present study population included individuals who were recruited using two strategies. First, if any of those recruited to the HDLI Investigation and Decision Committee through the Korea Environmental Industry & Technology Institute (KEITI) were pregnant, they were automatically included. Second, individuals voluntarily participated in the HD Extrapulmonary Injury Review Committee, and they submitted medical records of mothers and newborns exposed to HD during pregnancy, HD receipts, or photos of HD purchases. The study was conducted according to the declaration of Helsinki and the STROBE checklist. All participants signed informed consent for data review and use and the study protocol was approved by the Institutional Review Board of Dongtan Hallym University Sacred Heart Hospital (IRB No. 2019-12-14-001).

### Data collection

Data obtained from the medical records included maternal date of birth, maternal date of death, maternal date of starting HD use, maternal date of ending HD use, maternal date of symptom onset, neonatal birthday, neonatal birthweight, gestational age, and neonatal survival status within 28 days. All data were retrospectively investigated through medical records. The onset of clinical symptoms was defined as the day when the mother started complaining of symptoms other than pregnancy, especially respiratory symptoms, and neonatal death was defined as death within 28 days of birth.

### Calculated information

**Figure 1:**
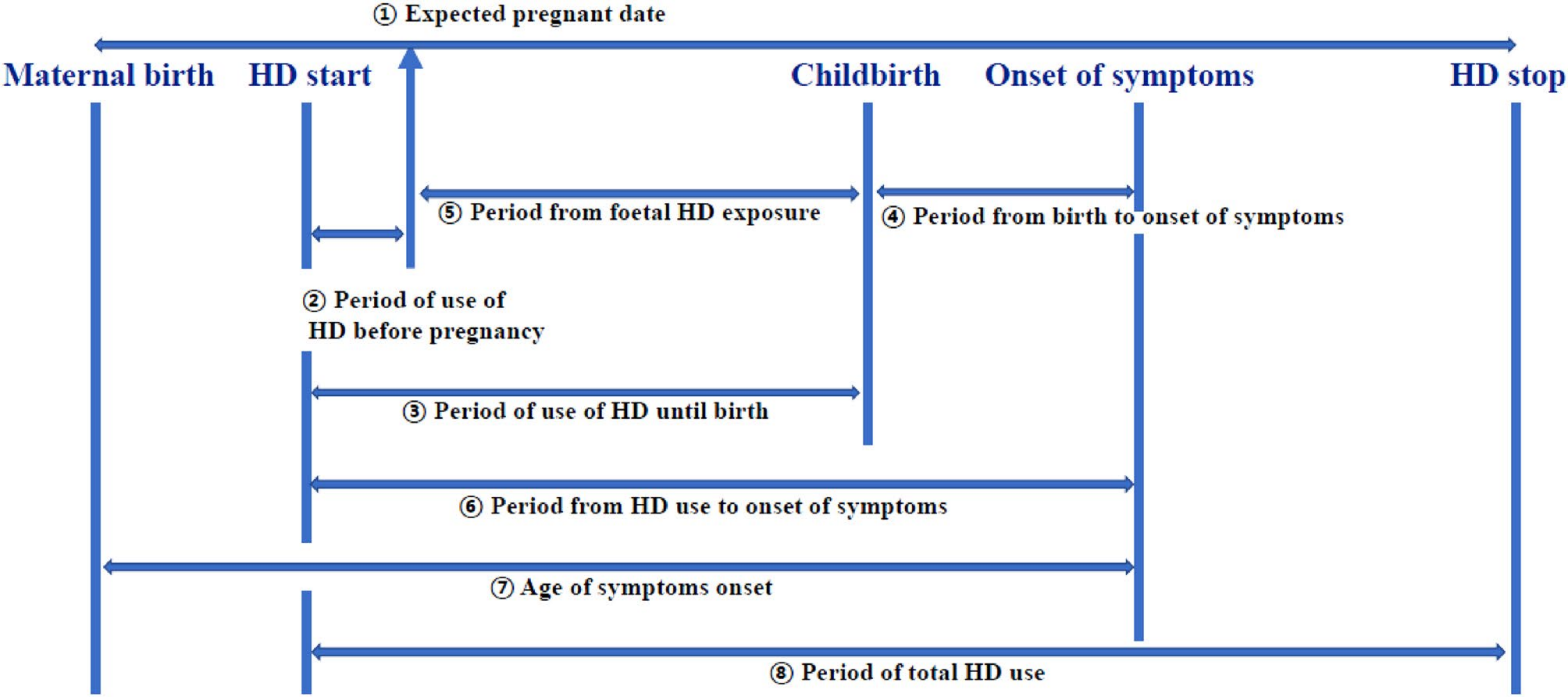
A schematic diagram of the period calculated using the collected information.

Based on the data obtained through medical records, the calculated information was obtained using the following calculation methods (Fig. 1):

① Expected pregnant date = neonatal birthday – Gestational age (weeks × 7 + days)
② Period of use of HD before pregnancy = Expected pregnant date – maternal date of HD starting (if the case of a negative value, it was converted to “0”)
③ Period of use of HD until birth = neonatal birthday – maternal date of HD starting
④ Period from birth to onset of symptoms = neonatal birthday – maternal date of symptom onset
⑤ Period of foetal HD exposure = neonatal birthday – expected pregnant date
⑥ Period from HD use to onset of symptoms
   = maternal date of symptom onset – maternal date of HD starting.
  (if the case of a negative value, the symptom was developed before childbirth)
⑦ Age of symptoms onset = maternal date of symptom onset – maternal date of birth
⑧ Period of total HD use = maternal date of HD ending – maternal date of HD starting
  or maternal date of death – maternal date of HD starting

It was divided into spring (March to May), summer (June to August), autumn (September to November), and winter (December to February) by the South Korean traditional definition. Trimester was divided into the first trimester (1–12 weeks), second trimester (13–26 weeks), and third trimester (27–40 weeks).

### Data analysis

Χ^2^-square tests or Fisher’s exact test were used to compare categorical data, and *t* tests were used to compare numerical data with normally distributed data, and Mann–Whitney U test were used with the data which were not followed a normal distribution. Differences between groups with estimated pregnancy dates in summer and non-summer seasons were compared. All statistical analyses were performed using SPSS version 25.0 (SPSS Inc., Chicago, IL, USA).

### Ethics approval and consent to participate

The present study protocol was reviewed and approved by the Institutional Review Board of Dongtan Hallym University Sacred Heart Hospital (IRB No. 2019-12-014-001).

## Results

### Clinical characteristics of participants

**Figure 2:**
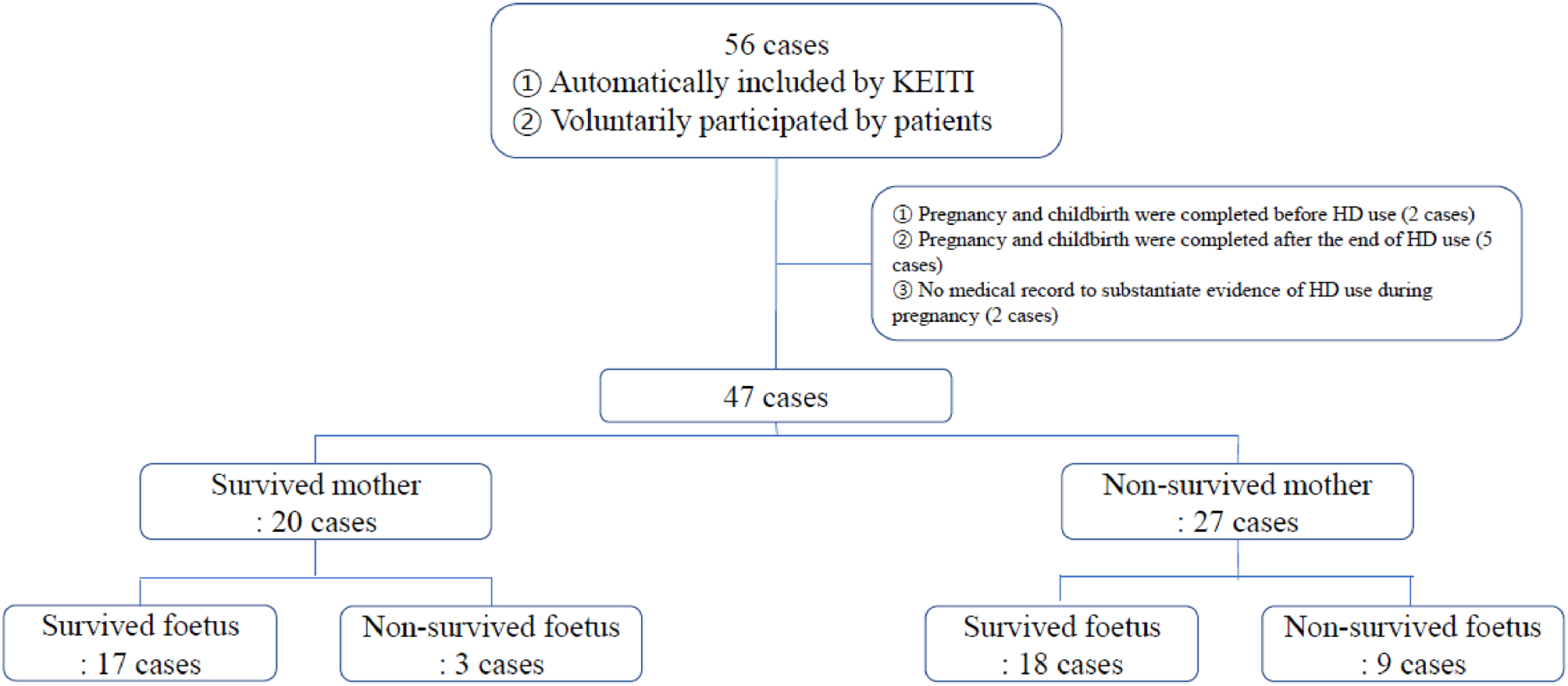
Study flowchart for the selection of subjects (n = 56).

Patients with overlapping HD use periods and pregnancy periods were included. A total of 56 foetal cases (42 cases in 2017, 12 cases in 2018, and 2 cases in 2019) received by KEITI were recruited. Of these, 47 cases were included, and 9 cases were excluded. The excluded cases were as follows: in 2 cases, pregnancy and childbirth were completed before HD use; in 5 cases, pregnancy and childbirth were completed after the end of HD use; and in 2 cases, there was no medical record to substantiate evidence of HD use during pregnancy (Fig. 2).

### Duration of HD use and maternal mortality

Of the 47 mothers, 20 mothers survived (42.6%), and 27 mothers died (57.4%). The period of HD use before pregnancy (227.2 ± 305.9 days vs. 416.9 ± 534.0 days, *p* = 0.036), and the period from HD use to onset of symptoms (589.6 ± 426.8 days vs. 776.1 ± 661.9 days, *p* = 0.037) were significantly shorter in surviving mothers (Table 1). The most common cause of maternal death was interstitial lung disease.

**Table 1:** Demographic findings based on maternal outcomes.

	Maternal alive (n = 20, 42.6%)	Maternal death (n = 27, 57.4%)	*P*
Age_OS (years)	32.0 ± 3.0	31.6 ± 3.3	0.949
Age_D		33.1 ± 2.3	
T_use (days)	644.4 ± 490.0	1019.5 ± 917.3	0.249
Until_birth (days)	431.2 ± 361.7	611.2 ± 551.8	0.131
Bef_pregnancy (days)	227.2 ± 305.9	416.9 ± 534.0	**0.036**
P_OS (days)	589.6 ± 426.8	776.1 ± 661.9	**0.037**
P_B_S (days)	164.6 ± 328.0	152.5 ± 376.3	0.579
F_Exp (days)	176.0 ± 86.6	195.4 ± 63.6	0.080
GA (days)	241.4 ± 65.3	236.2 ± 46.8	0.658
Bwt (g)	2293.9 ± 1072.5	2746.6 ± 982.3	0.299
Neonatal survival (numbers)	17	18	0.154
Estimated season of onset of pregnancy (numbers)			
Spring	5	5	
Summer	7	11	
Autumn	3	9	
Winter	5	2	
HD starting season (numbers)			
Spring	3	4	
Summer	3	6	
Autumn	6	15	
Winter	8	2	
HD starting trimester (numbers)			
1st Trimester	16	22	
2nd Trimester	3	5	
3rd Trimester	1	0	

**Age_OS,** age of onset of symptom; **Age_D**, age of death; **T_use**, period of total HD use; **Until_birth**, period of use of HD until birth; **Bef_Preg**, period of use of HD before pregnancy; **P_OS**, period from HD use to onset of symptoms; **P_B_S**, period from birth to onset of symptoms; **F_Exp**, period of foetal HD exposure; **GA**, gestational age; **Bwt**, birth weight; **HD**, humidifier disinfectant.

Significant values are in [bold].

### Comparison between maternal and neonatal outcomes by subgroups

Among the 20 surviving mothers, 17 (85.0%) neonates survived, and 3 (15.0%) neonates died. In the group of mothers who survived, younger gestational age (263.1 ± 26.2 days vs. 118.0 ± 90.1 days, *p* < 0.006), and smaller birth weight (2594.5 ± 856.0 days vs. 640.0 ± 99.0 days, *p* = 0.030) resulted in a higher the foetal mortality rate.

Among the 27 mothers who died, 18 (66.7%) neonates survived, and 9 (33.3%) neonates died. In the group of mothers who died, a shorter period from birth to onset of symptoms (255.4 ± 427.3 days vs. -53.3 ± 26.9 days, *p* < 0.001), and a shorter period of foetal HD exposure (221.5 ± 58.4 days vs. 143.0 ± 35.3 days, *p* = 0.010) resulted in a higher foetal mortality rate.

Among the 35 surviving newborns, 17 mothers survived (48.6%), and 18 mothers died (51.4%). In the group of mothers who survived, a shorter period of total HD use (647.1 ± 493.6 days vs. 1242.1 ± 952.4 days, *p* = 0.048), period of use of HD until birth (411.5 ± 319.2 days vs. 631.0 ± 497.4 days, *p* = 0.035), and period of use of HD before pregnancy (172.2 ± 249.3 days vs. 397.3 ± 479.6 days, *p* = 0.003) resulted in a higher maternal survival rate.

Among the 12 newborns who died, 3 mothers survived, and 9 mothers died. In the neonatal death group, symptoms appeared before birth on average across both subgroups of mothers (Table 2).

**Table 2:** Comparison between maternal and foetal outcomes by subgroups.

	Maternal alive (n = 20)			Maternal death (n = 27)			Neonatal alive (n = 35)			Neonatal death (n = 12)		
	NA (n = 17)	ND (n = 3)	*p*	NA (n = 20)	ND (n = 7)	*p*	MA (n = 17)	MD (n = 18)	*p*	MA (n = 3)	MD (n = 9)	*p*
Age_OS	32.0 ± 2.8	32.3 ± 4.9	0.711	31.0 ± 3.8	32.9 ± 1.3	0.198	32.0 ± 2.8	31.0 ± 3.8	0.338	32.3 ± 4.9	32.9 ± 1.3	0.926
T-use	647.1 ± 493.6	629.0 ± 575.2	0.958	1242.1 ± 952.4	574.4 ± 687.5	0.263	647.1 ± 493.6	1242.1 ± 952.4	**0.048**	629.0 ± 575.2	574.4 ± 687.5	0.782
Util_birth	411.5 ± 319.2	542.7 ± 636.9	0.546	631.0 ± 497.4	571.4 ± 679.0	0.973	411.5 ± 319.2	631.0 ± 497.4	**0.035**	542.7 ± 636.9	571.4 ± 679.0	0.782
Bef_Preg	186.5 ± 230.1	457.3 ± 609.5	0.299	411.1 ± 465.1	428.4 ± 683.2	0.762	172.2 ± 249.3	397.3 ± 479.6	**0.003**	457.3 ± 609.5	428.4 ± 683.2	0.781
P_OS	599.5 ± 407.4	533.7 ± 629.5	0.711	891.6 ± 642.9	545.2 ± 674.8	0.725	599.5 ± 407.4	891.6 ± 642.9	0.187	533.7 ± 629.9	545.2 ± 674.8	0.926
P_B_S	201.0 ± 343.9	− 41.7 ± 34.9	0.112	255.4 ± 427.3	− 53.3 ± 26.9	**< 0.001**	201.0 ± 343.9	255.4 ± 427.3	0.241	− 41.7 ± 34.9	− 53.3 ± 26.9	0.643
F_Exp	197.1 ± 88.8	85.3 ± 61.8	0.072	221.6 ± 58.4	143.0 ± 35.3	**0.010**	192.0 ± 81.4	221.6 ± 58.4	0.217	85.3 ± 61.8	143.0 ± 35.3	0.138
GA	263.1 ± 26.2	118.0 ± 90.1	**0.006**	262.6 ± 27.5	183.6 ± 28.8	0.700	263.1 ± 26.2	262.6 ± 27.5	0.694	118.0 ± 90.1	183.6 ± 28.8	0.195
	(37^+4^ ± 3^+5^)	(16^+6^ ± 12^+6^)		(37^+4^ ± 3^+6^)	(26^+2^ ± 4^+1^)		(37^+4^ ± 3^+5^)	(37^+4^ ± 3^+6^)		(16^+6^ ± 12^+6^)	(26^+2^ ± 4^+1^)	
Bwt	2594.5 ± 856.0	640.0 ± 99.0	**0.030**	2971.1 ± 780.1	950.0 ± 113.1	0.227	2594.6 ± 856.0	2971.1 ± 780.1	0.262	640.0 ± 99.0	950.0 ± 113.1	0.121

**NA**, neonatal alive; **ND**, Neonatal death; **Age_OS,** age of onset of symptom; **T_use**, period of total HD use; **Until_birth**, period of use of HD until birth; **Bef_Preg**, period of use of HD before pregnancy; **P_OS**, period from HD use to onset of symptoms; **P_B_S**, period from birth to onset of symptoms; **F_Exp**, period of foetal HD exposure; **GA**, gestational age; **Bwt**, birth weight; **HD**, humidifier disinfectant.

***Age_OS, T_use, P_OS****: Mann–Whitney test were used.*

Significant values are in [bold].

## Discussion

Park and Kwon^8^ investigated the maternal mortality rate from 2009 to 2014 using data from the Korea Statistical Office. According to the report, the maternal mortality rate (number of maternal deaths/number of women of reproductive age × 100,000) gradually increased to 0.45 in 2009, 0.55 in 2010, and 0.61 in 2011 but decreased to 0.37 in 2012, 0.38 in 2013, and 0.37 in 2014. This rate has gradually decreased and was confirmed to be 0.20 in 2021. This increase in maternal mortality from 2009 to 2011 was unusual, and after the sale of HD was banned in 2011, maternal mortality significantly declined. Thus, it was necessary to confirm the effects of HD on the mothers and foetuses of mothers who used HD.

Since November 2011, the Korean government has recruited cases to support HD victims through KEITI. In total, 7830 individuals have voluntarily reported thus far, of whom 1812 have died, with a mortality rate of 23.1%^9^. Among these cases, pregnant women were automatically classified and further investigated for harm to the foetus. As a result, both maternal (57.4%) and foetal (32.4%) mortality rates in mothers who used HD were confirmed to be higher than those reported nationwide.

When comparing maternal survival, the variables identified as significant were duration using HD before pregnancy and time to symptom onset (Table 1). The effect on the human body may vary depending on the following factors: spray amount of the humidifier; the distance between the humidifier and the human body; the concentration of HD used; the size of the space; and the degree of ventilation. Nevertheless, it was found that the duration of HD use increased significantly in the mother group who died regardless of the various factors mentioned above, suggesting that the use time before pregnancy is a factor that affects the human body.

In the comparison between maternal and neonatal outcomes by subgroups, the gestational age and birth weight in the neonatal survival group increased. Additionally, neonatal mortality increased with the onset of prenatal symptoms regardless of whether the mother survived. In particular, the average gestational age of the foetal death group in the maternal survival group corresponded to the beginning of the second trimester (16^+6^ ± 12^+6^), but the average gestational age of the foetal death group in the maternal death group corresponded to the end of the second trimester (26^+2^ ± 4^+1^) (Table 2). According to the “evolutionary nonself model” of immune tolerance that occurs during pregnancy, it is thought that the increased maternal survival in the group with foetal death in the early 2nd trimester suggests that when the critical value is exceeded in the mother's condition by using HD, it is recognized as a 'danger' state that exceeds immune tolerance, and a rapid immune response is activated^10^.

From this point of view, the role of the placenta should be considered. The placenta, through which medications pass and are delivered to the foetus, also serves to purify the substances that pass through. The placenta is a metabolically active tissue that responds to the maternal environment and can respond to perturbations during pregnancy by regulating the foetal supply of nutrients and oxygen as well as the secretion of hormones into the maternal and foetal circulation^11^. Importantly, due to significant functional differences in the placenta between the first two months of pregnancy (histiotrophic nutrition) and later in pregnancy (haemotrophic nutrition), there may be differences in the transplacental transfer of drugs^12^. In some cases, the mother’s inflammatory factors can cross the placenta and affect the foetus^13^. Inflammatory reactions in mothers can cause premature birth^14^ and foetal damage regardless of preterm birth^15^. A previous study on the effects of the persistent inhalation of chemicals by mothers has reported that birthweight decreases with increasing exposure to contaminants^16^.

When considering aspects of maternal mortality other than foetal death, it is necessary to consider the possibility that various effects of HD in addition to the role of the placenta may have had an effect. In terms of lung damage caused by the actions of these chemicals and various body reactions caused by inhalation, the subjects in the present study may have experienced direct effects of HD.

The effects of HDs on the mother that affect the foetus during pregnancy are as follows: (1) the increase in the maternal blood concentration of the drug directly affects the foetus as the drug passes through the placenta^17^; (2) metabolites that accumulate when drug concentrations increase in the mother’s body have effects on the foetus, such as inflammatory mediators^14^, hormones^18^, and toxins^19^; (3) the deterioration of the maternal physical condition caused by the medication (repair or damage to several organs) affects the foetus; and (4) implantation can be disrupted due to medication effects^20^.

The results, which were grouped by maternal survival, showed that survival was related to the total duration of HD use, especially the duration of prepregnancy HD use. These results may be associated with not only the direct effects of the causative agents but also the possibility that symptoms worsened rapidly due to the progression of damage to various maternal organs (especially the lungs) with continuous exposure. The longer duration of HD use was associated with a decreased maternal and foetal survival, which was consistent with reports that HD use exacerbates HDLI in a dose-dependent manner^7^.

### Study limitations

The present study had several limitations. First, the exact concentration and capacity of HD use as well as total seasonal consumption were not confirmed. The information was obtained from a blinded medical record, and because it was retrospectively reviewed, information to estimate the HD concentration (such as room size, average humidifier usage period, season of use, and ventilation status) was not obtained. To compensate for this limitation, converted dates were used, such as the date from the start of each event, seasonal classification, and trimester of exposure during pregnancy. Second, although we used objective information when collecting data, we cannot rule out recall bias over time. Third, the specific link between the effects of the maternal condition (such as hypoxia, hormones, inflammatory factors, and abnormalities in metabolic processes) on foetal growth were unknown. Fourth, even for a newborn with no abnormality at birth, it is difficult to confirm the long-term consequences of exposure during the foetal period and whether it affects future growth and development. Fifth, when mothers are exposed, it is necessary to consider the individual differences in maternal reactions. Sixth, regarding premature babies, the differences in medical technology and development over the past decade may have affected mortality. Finally, we could not compare nonpregnant women who had used HD or pregnant women who had not used HD in a similar age group because women who had no clinical symptoms had not used medical services or provided specific information about using HD. Because of this limitation, we studied these data as a nested case‒control study of women who had used HD during pregnancy.

### Study advantages

Despite these limitations, the present study is considered rare and contains important data on the relationship between maternal and foetal survival in response to HD exposure. Specifically, the present study provided data on foetal survival to small but persistent maternal inhalation exposure to chemical hazardous substances. Research to determine effects on the mother is contraindicated in research on substances that may be particularly harmful because they may affect the foetus as well as the mother. The present study epidemiologically confirmed maternal and foetal harm from unsafe chemical use. Although the increase in patients with lung damage due to the use of HD was limited to Korea, it is evaluated as an incident that raises awareness regarding the use of chemicals. The present study will be helpful for future research on the effects of environmental pollutants in the atmosphere, which have recently emerged.

## Conclusions

The present study analysed foetal survival during pregnancies exposed to HD. Shorter duration of HD use resulted in higher survival rate of mothers and increased gestational age of live foetuses. In addition, foetal mortality increased when clinical symptoms developed before birth.

## Data Availability

The datasets used and analysed during the current study are available from the corresponding author on reasonable request.

## Abbreviations

HD: Humidifier disinfectant
KEITI: Korea Environmental Industry & Technology Institute
PHMG: Polyhexamethylene guanidine phosphate
PGH: Oligo (2-(2-ethoxy) ethoxyethyl guanidinium
CMIT: Chloromethylisothiazolinone
MIT: Methylisothiazolinone
ILD: Interstitial lung disease
HDLI: Humidifier disinfectant lung injury
